# Senolytic treatment alleviates doxorubicin‐induced chemobrain

**DOI:** 10.1111/acel.14037

**Published:** 2024-01-15

**Authors:** Vivekananda Budamagunta, Ashok Kumar, Asha Rani, Sahana Manohar Sindhu, Yang Yang, Daohong Zhou, Thomas C. Foster

**Affiliations:** ^1^ Department of Neuroscience, McKnight Brain Institute University of Florida Gainesville Florida USA; ^2^ Genetics and Genomics Graduate Program, Genetics Institute University of Florida Gainesville Florida USA; ^3^ Department of Pharmacodynamics, College of Pharmacy University of Florida Gainesville Florida USA; ^4^ Department of Biochemistry and Structural Biology University of Texas Health Science Center at San Antonio San Antonio Texas USA

**Keywords:** chemobrain, cognition, inflammation, oxidative stress, senolytic NMDA receptor, transcription

## Abstract

Doxorubicin (Dox), a widely used treatment for cancer, can result in chemotherapy‐induced cognitive impairments (chemobrain). Chemobrain is associated with inflammation and oxidative stress similar to aging. As such, Dox treatment has also been used as a model of aging. However, it is unclear if Dox induces brain changes similar to that observed during aging since Dox does not readily enter the brain. Rather, the mechanism for chemobrain likely involves the induction of peripheral cellular senescence and the release of senescence‐associated secretory phenotype (SASP) factors and these SASP factors can enter the brain to disrupt cognition. We examined the effect of Dox on peripheral and brain markers of aging and cognition. In addition, we employed the senolytic, ABT‐263, which also has limited access to the brain. The results indicate that plasma SASP factors enter the brain, activating microglia, increasing oxidative stress, and altering gene transcription. In turn, the synaptic function required for memory was reduced in response to altered redox signaling. ABT‐263 prevented or limited most of the Dox‐induced effects. The results emphasize a link between cognitive decline and the release of SASP factors from peripheral senescent cells and indicate some differences as well as similarities between advanced age and Dox treatment.

## Graphical abstract

Doxorubicin (Dox) has limited brain access, and Dox treatments have nominal effects on brain genes that change with age. Nevertheless, Dox treatments mirrored other markers of aging; promoting peripheral cell senescence, pro‐inflammatory cytokines, microglial activation, a redox‐sensitive decrease in synaptic function, and impaired memory. The senolytic, ABT‐263, also has limited brain access. ABT‐263 treatments counteracted Dox effects to maintain memory function. The results highlight a link between peripheral cell senescence and the age‐related impairment of synaptic function required for memory.

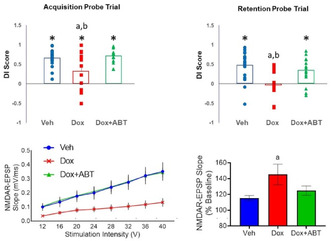

**Abbreviations**
ABT‐263: Navitoclax
BBB: Blood Brain Barrier
CA1: Cornus Amorus 1
CA3: Cornus Amorus 3
CDKN1A: Cyclin Dependent Kinase Inhibitor 1A
CDKN2A: Cyclin Dependent Kinase Inhibitor 2A
CXCL5: C‐X‐C motif Chemokine Ligand 5
DG: Dentate Gyrus
DI Score: Discriminatory Index Score
Dox: Doxorubicin
Dox+ABT: Animals Treated with Doxorubicin + ABT‐263
DTT: Dithiothreitol
EGF: Epidermal Growth Factor
EPSP: Excitatory Post Synaptic field Potentials
F344: Fisher 344
fEPSP: Functional Excitatory Post Synaptic field Potentials
GADD45G: Growth Arrest and DNA Damage inducible Gamma
G‐CSF: Granulocyte Colony‐Stimulating Factor
IA: Inhibitory Avoidance
Iba‐1: Ionized calcium‐Binding Adapter molecule 1
IFN‐γ: Interferon Gamma
IL12p70: Interleukin 12p70
IL13: Interleukin 13
IL17α: Interleukin 17 alpha
IL18: Interleukin 18
IL1α: Interleukin 1 alpha
IL1β: Interleukin 1 beta
IL2: Interleukin 2
IL4: Interleukin 4
IL5: Interleukin 5
IL‐6: Interleukin 6
IP10: C‐X‐C motif Chemokine Ligand 10 (CXCL10)
MCP1: Monocyte Chemoattractant Protein 1
MIP1α: Macrophage Inflammatory Protein 1 alpha
MIP2: Macrophage Inflammatory Protein 2
MMP3: Matrix Metallopeptidase 3
MWM: Morris Water Maze
NMDA: N‐methyl‐D‐aspartate
NMDAR: N‐methyl‐D‐aspartate Receptor
SASP: Senescence Associated Secretory Phenotype
TGFB3: Transforming Growth Factor Beta 3
TNFSF11: Tumor Necrosis Factor Super Family member 11
TNFα: Tumor Necrosis Factor alpha
Veh: Vehicle

## 1 INTRODUCTION

Chemobrain is an umbrella term for chemotherapy‐induced cognitive impairments (Janelsins et al., 2014). In the United States, ~2 million new cases of cancer are diagnosed annually, and ~75% of patients who receive chemotherapy report signs of cognitive changes associated with chemobrain during the duration of the treatment, with ~35% of the patients reporting cognitive impairment lasting months or years following treatment (Janelsins et al., 2014). Approximately 5% of the US population is comprised of cancer survivors, and their number is projected to grow by over 30% before the end of this decade (Miller et al., 2019). With a dramatic increase in the survival rate of cancer patients (Dulskas et al., 2020; Harding et al., 2020; Kim et al., 2012; Manegold & Thatcher, 2007; Sun et al., 2014), chemobrain has become an important side‐effect to be addressed, as it significantly impacts the quality of life of cancer survivors (Du et al., 2021; Eide & Feng, 2020; Henderson et al., 2019).

Despite being widely recognized as one of the most common side effects of chemotherapy, there is a lack of a comprehensive understanding of underlying mechanisms that fundamentally drive chemobrain. Not surprisingly, many have described an accelerated aging process in cancer survivors (Sulicka‐Grodzicka et al., 2021; Wang et al., 2021). Similar to aging, chemotherapy is associated with an increased prevalence of conditions such as frailty (Ness et al., 2013; Ness & Wogksch, 2020; Smitherman et al., 2020), bone loss (Drake, 2013; Haddy et al., 2001; Jeong et al., 2019; Mahon, 1998; Ramin et al., 2018; Tuck et al., 2013), secondary cancer incidence (Demaria et al., 2017; Demoor‐Goldschmidt & de Vathaire, 2019; Morton et al., 2014), pulmonary fibrosis (Carver et al., 2007; Mertens et al., 2002), cardiovascular pathologies (Aleman et al., 2014; Armenian et al., 2016; Keegan et al., 2018; Okwuosa et al., 2017), chronic sterile inflammation (Bower et al., 2002; Cupit‐Link et al., 2017; LaVoy et al., 2016), and insulin resistance (Baker et al., 2013; Steinberger et al., 2012) in cancer survivors. The mechanisms for chemobrain include induction of cellular senescence, increased oxidative stress, and inflammation (Cardoso et al., 2020; Chen et al., 2007; Conklin, 2004; Gibson et al., 2019; Hurria et al., 2016; Hutchinson et al., 2012; Vyas et al., 2014; Wang et al., 2015). Chemotherapeutic agents like doxorubicin (Dox) have been employed as a model of aging and age‐related cognitive decline (Abdelgawad et al., 2023; Bagnall‐Moreau et al., 2019; Sun et al., 2022).

Cellular senescence is a phenomenon characterized by a cell undergoing stable cell cycle arrest upon encountering an intrinsic or extrinsic stressor (Campisi & d'Adda di Fagagna, 2007). A cardinal feature of cellular senescence is the senescence‐associated secretory phenotype (SASP) (Aird et al., 2016; Coppe et al., 2010; Hernandez‐Segura et al., 2018). SASP is comprised of a wide variety of secretory proteins including inflammatory cytokines, growth factors, immune modulators, and tissue remodeling proteins (Basisty et al., 2020; Budamagunta et al., 2023; Coppe et al., 2010; Schafer et al., 2020). As such, SASP acts as one of the most important mediators for a wide variety of effects caused by cellular senescence (Budamagunta et al., 2021; Coppe et al., 2010; Demaria et al., 2014; Ogata et al., 2021; Oubaha et al., 2016). Recent research has demonstrated that senolytics, a class of small molecules that selectively eliminate senescent cells, preserves cognition during aging (Budamagunta et al., 2023). Moreover, senolytic treatment can also improve cognitive impairment associated with irradiation (Fielder et al., 2022; Tarantini et al., 2021; Yabluchanskiy et al., 2020). Given the understanding that chemotherapeutic agents like Dox can induce senescence and the role of senescent cells in cognitive impairment, we hypothesized that a senolytic treatment could alleviate the effects of chemobrain in Dox‐treated rats.

The current study confirms that Dox treatment has some effects similar to aging, increasing markers of cellular senescence in the periphery, including an increase in SASP pro‐inflammatory cytokine/chemokine levels in the blood. Dox treatment was also associated with loss of blood–brain barrier (BBB) integrity, increased microglial activation, a redox‐mediated decrease in N‐methyl D‐aspartate receptor (NMDAR) function, and impairment in NMDAR‐dependent cognitive function. Importantly, these adverse effects of Dox were greatly diminished by treatment with ABT‐263 (navitoclax), an established senolytic agent (Chang et al., 2016; Dookun et al., 2020; Gonzalez‐Gualda et al., 2020; He et al., 2020; Kirkland & Tchkonia, 2020). The results emphasize a link between chemotherapy‐induced cognitive decline and peripheral inflammation, enhancing oxidative stress in the brain, to modify synaptic function.

## 2 RESULTS

### 2.1 ABT‐263 reduced Dox‐mediated peripheral senescence burden and inflammation

**FIGURE 1:**
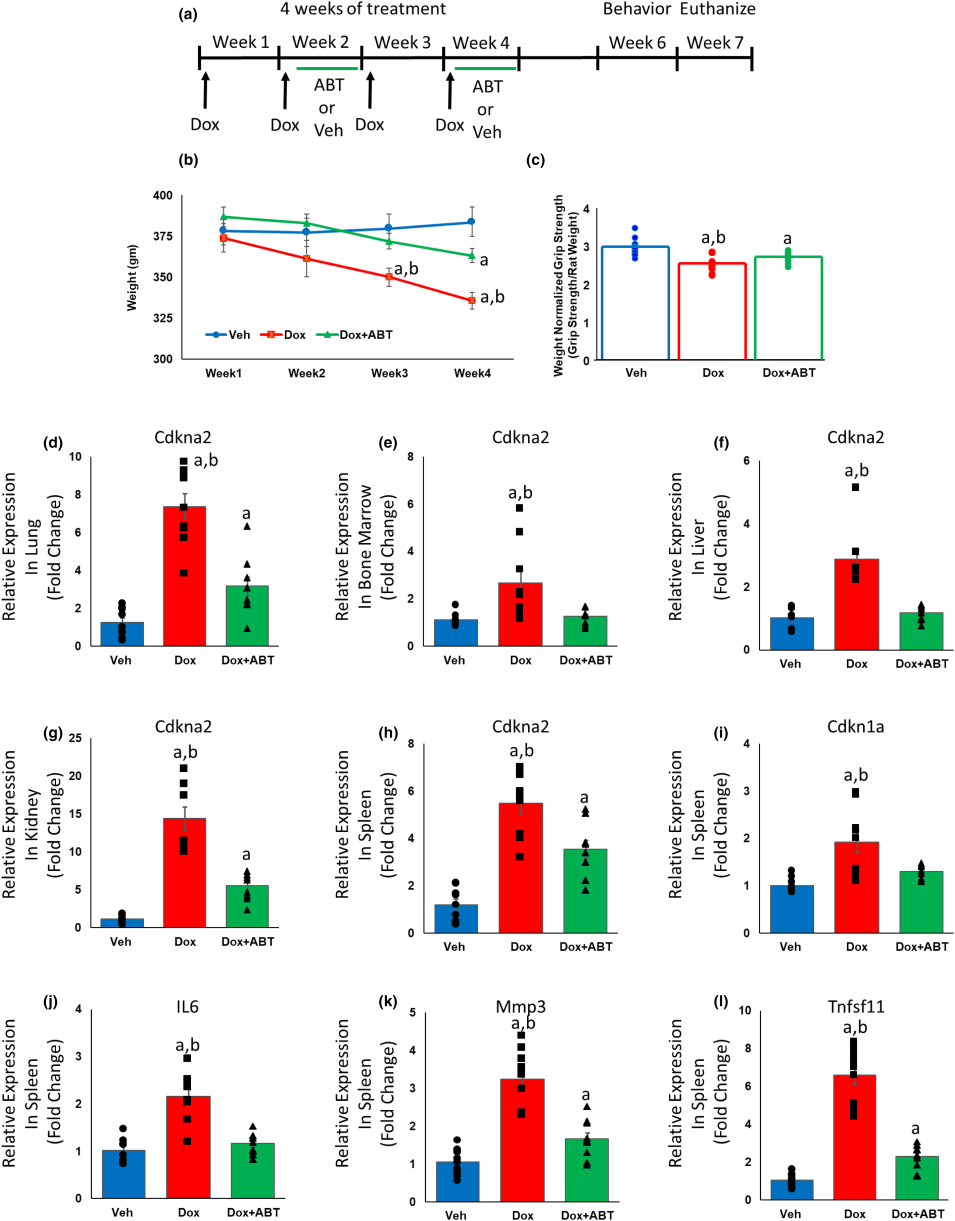
Dox treatment increased and ABT‐263 treatment decreased expression of senescent and SASP genes in the periphery. (a) Time course of treatment, starting at 6 months of age. For the two groups receiving Dox treatment, 2 mg/kg dose of Dox was administered by injection once a week (arrows) for 4 weeks, while the group receiving the senolytic treatment received a 12 mg/kg dose of ABT‐263 by oral gavage for 5 consecutive days every other week (Weeks 2 and 4, green bars). The vehicle group also received oral gavage of vehicle for 5 consecutive days every other week. Starting 2 weeks after the last dose of Dox and 1 week after the last ABT‐263 treatment, animals were behaviorally characterized (*n* = 22 for Veh, *n* = 13 for Dox and *n* = 14 for Dox + ABT). (b) Change in body weight over the course of the study (*n* = 14 for Veh, *n* = 13 for Dox and *n* = 14 for Dox + ABT). (c) Grip strength normalized to weight (*n* = 14 for Veh, *n* = 13 for Dox and *n* = 14 for Dox + ABT). For a–c, data represented as mean ± SEM; a = significantly different (*p* < 0.05) from Veh and b = significantly different (*p* < 0.05) from Dox + ABT. Relative to Veh and Dox + ABT groups, treatment with Dox alone increased the expression level of *Cdkn2a* in (d) lung, (e) bone marrow, (f) liver, (g) kidney, and (h) spleen. Relative to Veh and Dox + ABT groups, treatment with Dox alone increased the expression level of (i) *Cdkn1a*, (j) IL‐6, (k) *Mmp3*, and (l) *Tnfsf11* in spleen. Error bars denote SEM (*n* = 9 per group). a = significantly different (*p* < 0.05) from Veh, b = significantly different (*p* < 0.05) from Dox + ABT.

For a subset of animals (Veh = 14, Dox = 13, Dox + ABT = 14), body weight was tracked across the 4 weeks of treatment. A repeated measures ANOVA on the body weight across the 4 weeks yielded a significant effect of treatment [*F*_(2, 38)_ = 3.50; *p* < 0.05] and time [*F*_(3, 114)_ = 23.72; *p* < 0.0001], and an interaction [*F*_(6, 114)_ = 11.37; *p* < 0.0001]. The observed significance was mainly due to a reduction in weight of the Dox‐treated animals over time relative to Veh‐treated animals, which was attenuated by the treatment with ABT‐263 (Figure 1a,b). An ANOVA on the weight normalized grip strength also indicated that there was a significant effect of treatment [*F*_(2, 38)_ = 20.83; *p* < 0.0001] on the animals' performance. Post hoc comparisons revealed that each group was significantly different from the other groups, with the Dox group having the lowest grip strength, which was partially recovered by treatment with ABT‐263 (Figure 1c).

Dox treatment increased the expression of the senescence gene, *Cdkn2a*, as reported previously (Lopez‐Dominguez et al., 2021; Sun et al., 2022). To determine whether ABT‐263 reduced peripheral senescence accumulation associated with Dox treatment, we examined the levels of senescence‐associated genes across the three groups using RT‐PCR (Veh = 9, Dox = 9, Dox + ABT = 9). A significant (*p* < 0.005) effect of treatment was observed for the senescence marker gene, *Cdkn2a*, in all tissues examined (spleen, lung, bone marrow, liver, and kidney) (Figure 1d–h). Post hoc analysis indicated that, relative to Veh and Dox + ABT, *Cdkn2a* was elevated in the Dox group. In some cases (lung, kidney, and spleen), expression of *Cdkn2a* for Dox + ABT was intermediate between Dox and Veh, such that expression was elevated relative to Veh and decreased relative to Dox treatment. In bone marrow and liver, no difference in *Cdkn2a* was observed between Veh and Dox + ABT. Similarly, expression of other senescence associated genes, *Cdkn1a*, *IL‐6*, *Mmp3*, and *Tnfsf11*, were significantly (*p* < 0.0001) increased in the spleen in the Dox group relative to Veh and the expression pattern for Dox + ABT treatment was intermediate, elevated relative to Veh for *Mmp3*, and *Tnfsf11* and not different from Veh for *Cdkn1a* and *IL‐6* (Figure 1i–l).

The plasma was examined for an array of cytokines/chemokines (*n* = 6 per group) (Table 1). In general, Veh and Dox + ABT were not different from each other. Dox treatment increased inflammatory markers relative to Veh and Dox + ABT (*p* < 0.05) for fractalkine, G‐CSF, IL‐1β, IL‐5, IL‐13, IL‐18, CXCL5, MCP‐1, MIP‐1α, and RANTES. The level of IP‐10 was elevated in the Dox group relative to Veh, and IP‐10 levels for the Dox + ABT group were intermediate and not different from the other groups.

**TABLE 1:** Treatment effect on chemokines, cytokines, and hormone concentration.

Analyte	Veh	Dox	Dox + ABT
Cxcl5	498.49 ± 105.16	1349.93 ± 344.88^a^ ^,^ ^b^	593.50 ± 61.06
EGF	324.14 ± 74.77	278.62 ± 96.29	256.13 ± 93.61
Eotaxin	5.67 ± 1.54	11.31 ± 1.92^a^ ^,^ ^b^	8.62 ± 3.14
Fractalkine	77.80 ± 13.06	119.99 ± 15.15^a^ ^,^ ^b^	70.40 ± 6.33
G‐CSF	4.31 ± 1.57	42.04 ± 10.06^a^ ^,^ ^b^	13.91 ± 9.71
IFNγ	162.66 ± 93.49	344.62 ± 71.24	142.87 ± 51.44
IL‐1α	33.73 ± 8.26	89.67 ± 17.04	60.89 ± 40.16
IL‐1β	42.36 ± 9.09	167.26 ± 45.86^a^ ^,^ ^b^	52.55 ± 9.52
IL‐2	117.02 ± 12.79	264.09 ± 110.80^a^ ^,^ ^b^	125.77 ± 13.82
IL‐5	79.78 ± 11.19	207.74 ± 55.45^a^ ^,^ ^b^	98.27 ± 12.69
IL‐4	23.06 ± 5.66	47.93 ± 14.78	34.15 ± 2.63
IL‐6	475.8 ± 113.35	891.79 ± 149.69	549.08 ± 335.66
IL‐12p70	289.97 ± 34.54	354.36 ± 149.51	272.35 ± 53.18
IL‐13	11.93 ± 5.48	28.96 ± 3.78^a^ ^,^ ^b^	8.31 ± 2.53
IL‐17α	33.00 ± 4.97	90.15 ± 33.08	37.35 ± 5.88
IL‐18	387.79 ± 28.84	832.12 ± 198.73^a^ ^,^ ^b^	331.99 ± 22.12
IP‐10	243.01 ± 29.83	414.39 ± 40.51^a^	309.41 ± 38.12
Leptin	12121.17 ± 4558.92	20181.10 ± 5467.45	27347.09 ± 7752.62
MCP‐1	594.18 ± 119.04	5081.39 ± 2351.15^a^ ^,^ ^b^	793.72 ± 102.53
IMP‐1α	19.68 ± 3.85	64.40 ± 18.59^a^ ^,^ ^b^	20.26 ± 1.12
MIP‐2	21.65 ± 3.84	40.58 ± 8.40	28.66 ± 10.79
RANTES	851.98 ± 219.69	5085.44 ± 2273.75^a^ ^,^ ^b^	1421.36 ± 217.06
TNFα	5.27 ± 1.22	18.42 ± 8.07	6.90 ± 1.17
VEGF	71.55 ± 25.53	135.39 ± 42.45	44.85 ± 16.61

*Note*: Data are represented as mean ± SEM (*n* = 3–6).

^a^ *p* <0.05 vs. Veh.

^b^ *p* <0.05 vs. Dox + ABT.

### 2.2 Behavioral impairment following Dox treatment was attenuated by ABT‐263 treatment

**FIGURE 2:**
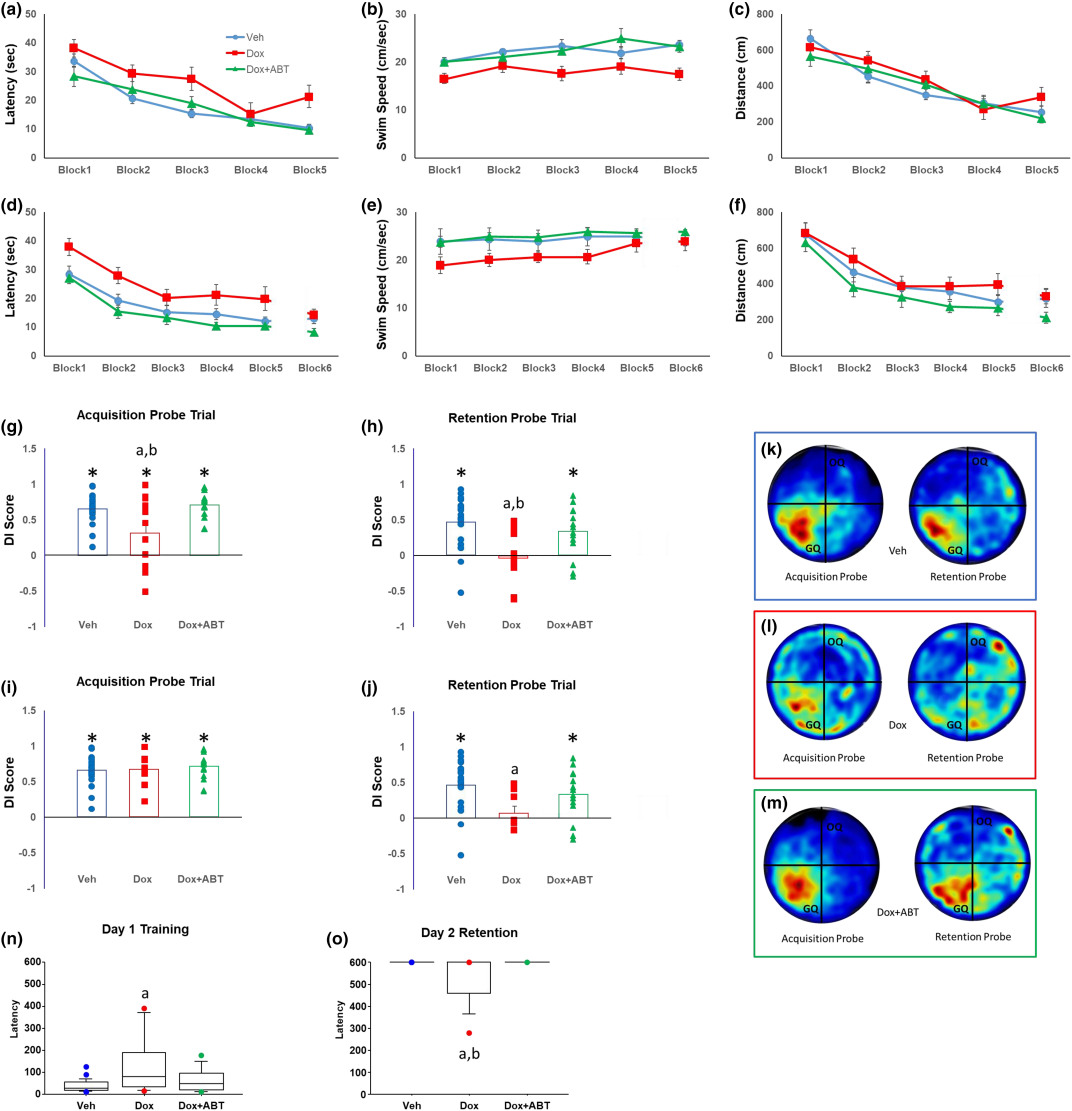
ABT‐263 treatment ameliorated Dox‐induced impairments in cognition. (a, d) latency, (b, e) swim speed and (c, f) escape distance for each training block for the cue discrimination a–c and spatial discrimination d–f tasks. The data are expressed as Mean ± SEM. Discrimination index (DI score) for the (g) acquisition and (h) retention probe trials on the watermaze. (i, j) Same data as g–h, except that data for the Dox group were limited to those that performed above chance (DI score >0) during acquisition testing. (k–m) Group mean heat maps for the animal's position during the acquisition and retention probe trials. (n, o) Box plots for latency to enter the dark compartment of inhibitory avoidance during (n) Day 1 training and (o) Day 2 retention testing (*n* = 22 for Veh, *n* = 13 for Dox and *n* = 14 for Dox + ABT); Data represented as mean ± SEM; a = significantly different (*p* < 0.05) from Veh, b = significantly different (*p* < 0.05) from Dox + ABT. Asterisks indicate a significant difference (*p* < 0.05) from chance performance (g–j).

As plasma cytokines/chemokines can cross the BBB to impact CNS functioning (Aluise et al., 2010a; Budamagunta et al., 2023), we behaviorally characterized the animals to assess their cognitive function. A repeated measures ANOVA on the escape latency (time to reach the escape platform) across training blocks for the cue discrimination task indicated a significant effect of training [*F*_(4, 184)_ = 36.53; *p* < 0.0001] and treatment [*F*_(2, 46)_ = 7.70; *p* < 0.05] in the absence of an interaction. Post hoc tests indicated poorer performance by the Dox group relative to the other two groups (Figure 2a). Latency differences were due, at least in part, to slower swim speed for Dox animals. An ANOVA for swim speed across trials indicated an effect of training [*F*_(4, 184)_ = 3.73; *p* < 0.01] with increased swim speed over the course of training and an effect of treatment [*F*_(2, 46)_ = 12.13; *p* < 0.0001] in the absence of an interaction (Figure 2b). Post hoc tests indicated that the Dox group had a slower swim speed relative to Veh and Dox + ABT. In contrast, a repeated measures ANOVA across training blocks for the distance to escape (Figure 2c), yielded a significant effect of training [*F*_(4, 184)_ = 35.14; *p* < 0.0001] in the absence of a treatment effect. The results indicate that Dox treatment decreased swim speed but did not disrupt the ability to acquire the procedural aspects of the task. Furthermore, the decline in swim speed was ameliorated by ABT‐263 treatment.

Three days following the cue discrimination task, a 1‐day version of the spatial water maze task was performed. A repeated measures ANOVA confirmed a significant effect across training blocks [*F*_(5, 230)_ = 31.10; *p* < 0.0001] and treatment [*F*_(2, 46)_ = 9.94; *p* < 0.0005] on the latency to escape (Figure 2d). Again, post hoc tests indicated a longer latency for the Dox group compared to the other two groups. Examination of swim speed confirmed an increase in swim speed over the course of learning [*F*_(5, 230)_ = 5.31; *p* < 0.001] and a treatment effect [*F*_(2, 46)_ = 9.10; *p* < 0.001], with a significant interaction between treatment and training [*F*_(10, 230)_ = 2.13; *p* < 0.05] due to a slower swim speed for Dox‐treated animals relative to the other groups (Figure 2e). A repeated measures ANOVA on the distance to platform yielded a significant effect across training blocks [*F*_(5, 230)_ = 22.24; *p* < 0.0001] and a treatment effect [*F*_(2, 46)_ = 3.54; *p* < 0.05] in the absence of an interaction. Post hoc comparisons indicated that Dox‐treated animals exhibited an increase in pathlength relative to the Dox + ABT group (Figure 2f).

The discrimination index (DI) scores for the acquisition probe trial exhibited a significant treatment effect [*F*_(2, 46)_ = 6.78; *p* < 0.005] and post hoc analysis indicated poorer performance by the Dox treatment group relative to Veh and Dox + ABT. However, one group *t*‐tests on the DI scores of each of the three groups indicated that all groups were significantly above chance (DI score = 0) (Figure 2g). Examination of the DI scores for the 24‐h retention probe trial indicated a significant treatment effect [*F*_(2, 46)_ = 8.11; *p* < 0.005] due to lower DI scores for the Dox‐treated group relative to Veh and Dox + ABT. One group *t‐*tests on the retention probe trial DI scores for the three groups indicated that the DI scores were above chance only for the Veh and Dox + ABT treatment groups (Figure 2h), indicating a memory deficit for the Dox group. It is possible that the observed memory deficit was due to impaired learning for the Dox group, as we observed a significant deficit in the DI score of Dox animals for the acquisition probe trial. Therefore, to test for a memory deficit, we excluded five animals from the Dox group, which did not exhibit learning during the acquisition probe trial (i.e., DI ≤0 for the acquisition probe trial) and reevaluated performance. Using this exclusion criterion resulted in no group differences in DI scores for the acquisition trial (Figure 2i); however, a treatment effect was still observed for the retention trial [*F*_(2, 41)_ = 3.92; *p* < 0.05] due to decreased retention in the Dox group relative to the Veh group. Moreover, the retention DI scores for the Dox group continued to not be different from chance (Figure 2j). The results strongly indicate an impairment in the memory retention for the Dox‐treated animals, which was ameliorated by ABT‐263 treatment.

For the inhibitory avoidance task, a Kruskal–Wallis test on the data pertaining to the latency of the rats to enter the dark chamber on the Day 1 training day yielded a significant effect of treatment (H = 7.59; *p* < 0.05) (Figure 2n). Post hoc Mann–Whitney *U* tests indicated an increased latency for the Dox group relative to Veh, with no difference between Dox + ABT and the other two groups. A Kruskal–Wallis test for the Day 2 retention test latency to enter the dark chamber showed a significant effect of treatment (H = 11.79; *p* < 0.005). Post hoc Mann–Whitney *U* tests yielded a significant difference between Dox, and both Veh and Dox + ABT groups (Figure 2o). The difference was due to ~30% of the Dox‐treated animals (4 of 13 animals) re‐entering the dark chamber at some point during the retention testing while none of the Veh or the Dox + ABT animals entered the dark chamber (Figure 2o). Together, these results indicate that Dox treatment decreased swim speed and impaired the retention of spatial/contextual memory and these behavioral deficits were alleviated by the senolytic treatment with ABT‐263.

### 2.3 Dox‐induced impairment of NMDAR synaptic function was ameliorated by ABT‐263 treatment

**FIGURE 3:**
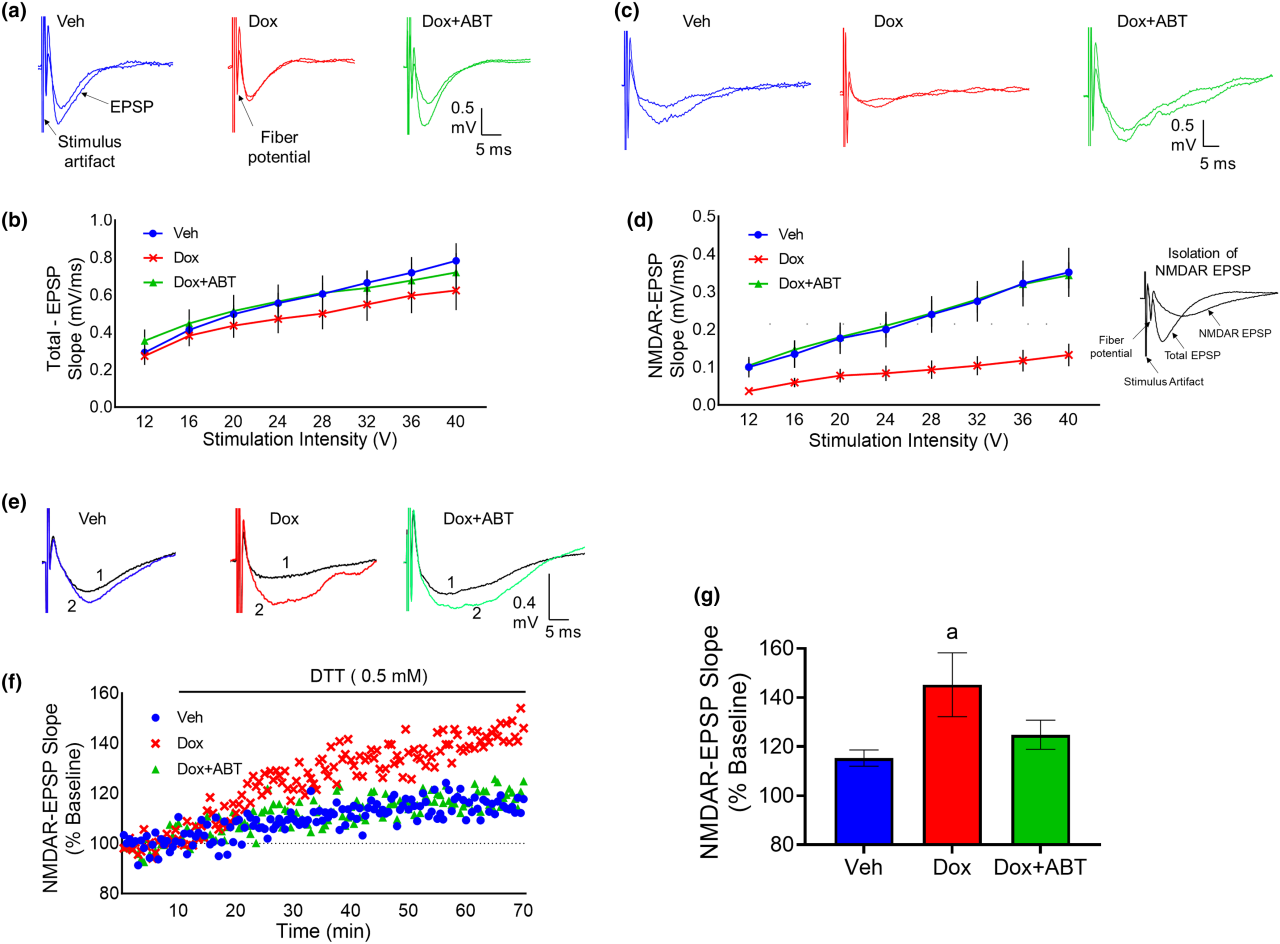
ABT‐263 treatment preserved synaptic function in the hippocampus. (a) Examples for the total synaptic responses for stimulation intensity at 12 and 20 V for the three groups. (b) Input/output curve of the mean ± SEM slope of total excitatory postsynaptic field potentials recorded from hippocampal CA3‐CA1 synapses. (c) Examples for the NMDAR synaptic responses for stimulation intensity at 16 and 20 V for the three groups. (d) Input/output curve of the mean ± SEM slope of NMDAR synaptic field potentials recorded from hippocampal CA3‐CA1 synapses. (e) Examples for the NMDAR synaptic response before (1) and 1 h after (2) application of DTT. (f) Time course of the growth of the NMDAR‐mediated component of the EPSP during application of DTT (bar). Each point represents the mean relative to the baseline. (g) Mean ± SEM growth of the NMDAR‐mediated component of the EPSP, 1 h after application of DTT. Data are represented as mean ± SEM. *n* = 10/5 slices/animals for Dox; *n* = 8/4 slices/animals for Veh and *n* = 7/4 slices/animals for Dox + ABT. a = significantly different (*p* < 0.05) from Veh.

Electrophysiological characterization of hippocampal slices was carried out to assess the effects of Dox on the total synaptic response (Figure 3a,b) and the NMDAR component of the synaptic response (Figure 3c,d). A repeated measures ANOVA on the input/output curve for the slope of the total synaptic response indicated a significant effect of stimulation intensity on the response [*F*_(7, 147)_ = 60.89; *p* < 0.0001] in the absence of a treatment effect or interaction (Figure 3b). In contrast, an effect of stimulation intensity [*F*_(7, 161)_ = 101.63; *p* < 0.0001] and treatment [*F*_(2, 23)_ = 6.53; *p* < 0.01] and an interaction of stimulation intensity and treatment [*F*_(14, 161)_ = 8.30; *p* < 0.0001] was observed for the NMDAR component of the synaptic response (Figure 3d). Treatment effects were due to a decrease in the response of Dox group relative to Veh and Dox + ABT groups.

Previous work indicates that a decrease in NMDAR function can result from altered redox signaling due to an increase in oxidative stress associated with aging and inflammation (Bodhinathan et al., 2010; Foster, 2019; Kumar, Yegla, et al., 2018). To test this idea, the stimulation intensity was adjusted to evoke an NMDAR synaptic response ~50% of maximum (Figure 3e). After recording a stable baseline, the reducing agent dithiothreitol (DTT, 0.5 mM) was bath applied and the response was followed for 1 h (Figure 3f). Figure 3g shows the mean increase in the NMDAR‐mediated synaptic response at the end of 1 h. An ANOVA indicated a tendency (*p* = 0.09) for an effect of treatment on the growth of the response to DTT. Post hoc tests indicated that DTT significantly increased the response for the Dox group (*n* = 10/5 slices/animals) relative to the Veh group (*n* = 8/4 slices/animals), which was attenuated by the treatment with ABT‐263 (*n* = 7/4 slices/animals). These results demonstrate that the Dox treatment‐induced impairment in NMDAR synaptic function is linked to redox signaling and can be alleviated by the ABT‐263 senolytic treatment.

### 2.4 The effect of Dox on the transcriptome was attenuated by ABT‐263 treatment

We employed next‐generation sequencing to examine Dox‐induced alterations of gene expression in the dentate gyrus (DG) of the hippocampus. Statistical filtering (*p* < 0.01) for differences in gene expression indicated that, compared to the Veh group (*n* = 10), the Dox group (*n* = 8) exhibited 325 genes that increased and 365 genes that decreased expression. These 690 genes were used to compare the Veh group (*n* = 10) and Dox + ABT group (*n* = 8). Statistical filtering (*p* < 0.01) for differences in gene expression revealed that combining Dox with ABT‐263 attenuated the effect of Dox, as 82% of the genes influenced by Dox were not different from the Veh group when ABT‐263 was included in the treatment.

**FIGURE 4:**
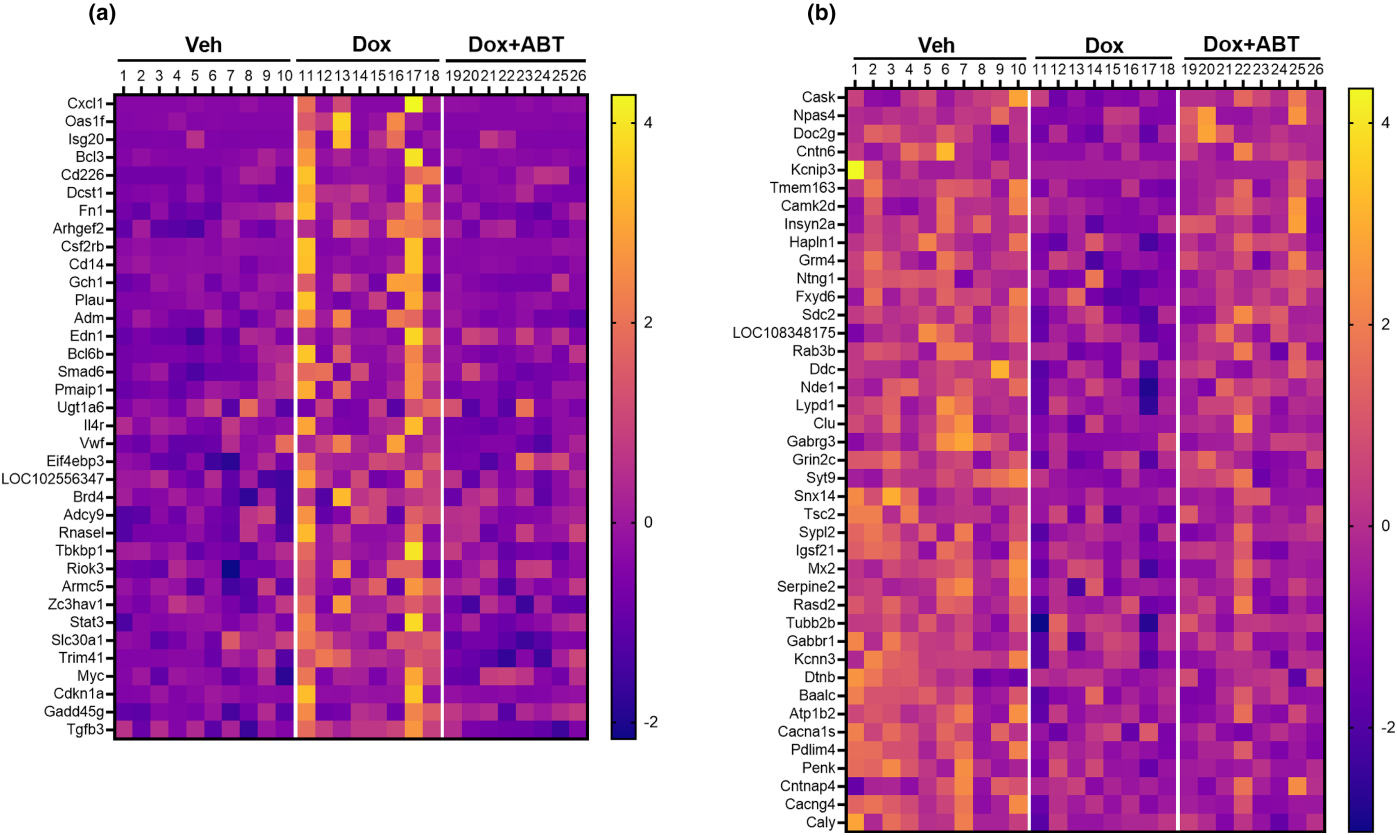
Effects of treatment on DG gene expression. Heatmap of expression patterns of DG genes under conditions of Veh, Dox, and Dox + ABT. (a) Heatmap of 37 genes that increased expression (*p* < 0.01) in Dox relative to Veh groups and were linked to lipopolysaccharide response, immune effector response or cellular senescence. (b) Heatmap of 41 synaptic component genes that decreased expression (*p* < 0.01) in Dox relative to Veh groups. Data are represented as *z*‐scores, normalized relative to the average expression across all animals (*n* = 10 for Veh; *n* = 8 for Dox and *n* = 8 for Dox + ABT).

Nondirected gene cluster analysis for genes that increased expression in the Dox group (*n* = 8), relative to the Veh group (*n* = 10), indicated enrichment for categories involved in detoxification including glucuronidation, cellular response to xenobiotic stimulus, metabolism of xenobiotics by cytochrome P450, positive regulation of cell death, and nitric oxide transport. Decreased expression was linked to nervous system development, neurogenesis, and myelination. Notably absent was significant gene clustering for categories normally linked to aging including increased expression of genes involved in regulation of immune response, oxidative stress, cellular senescence, and decreased expression for synaptic component genes. In many cases, there were several genes altered within these categories; however, the total number did not reach the level of significance for nondirected analysis. For example, Dox treatment was associated with increased expression of 33 genes involved in the lipopolysaccharide or immune effector response and 4 genes involved in cellular senescence (*Cdkn1a*, *Myc*, *Gadd45g*, *Tgfb3*). For these 37 genes, 27 were significantly (*p* < 0.05) upregulated in the Dox group relative to the Dox + ABT group and expression was similar between the Dox + ABT group and Veh group (Figure 4a). Likewise, for 41 synaptic component genes that were decreased in the Dox group relative to the Veh group, the inclusion of ABT‐263 muted the effect of Dox treatment (Figure 4b).

### 2.5 Dox‐induced neuroinflammation and impairment of BBB integrity was mitigated by ABT‐263 treatment

**FIGURE 5:**
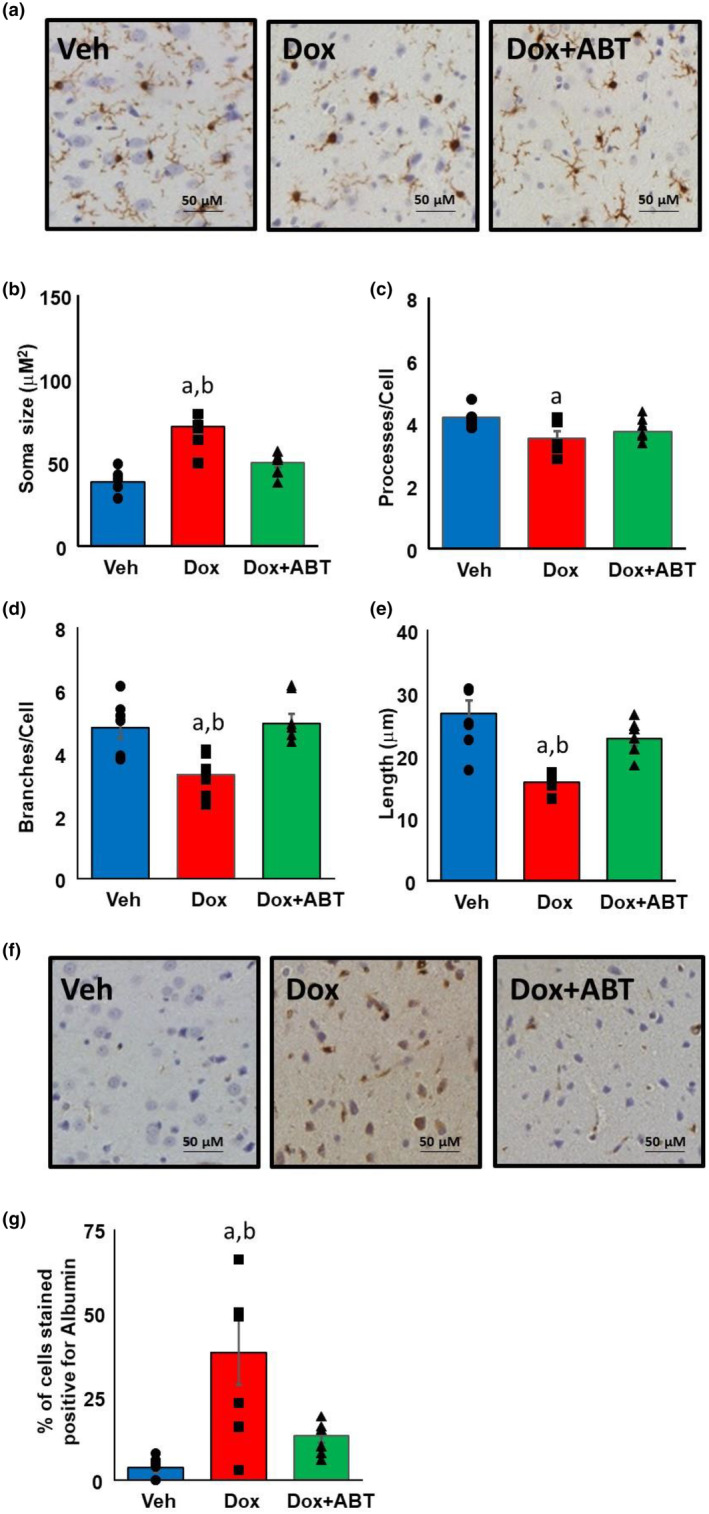
Dox‐induced microglial activation was reduced by ABT‐263. (a) Representative cortex sections immunohistologically stained for Iba‐1. Mean ± SEM for microglial morphological parameters (b) soma size, (c) processes per cell, (d) branches per cell, and (e) the average length of the process. (f) Representative cortex sections immunohistologically stained for albumin. (g) Bars represent mean ± SEM of proportion of cells stained positive for albumin. *n* = 6 per group; a = significantly different (*p* < 0.05) from Veh, b = significantly different (*p* < 0.05) from Dox + ABT.

Results from previous studies established that Dox administration induces morphological changes associated microglial activation (Chunchai et al., 2022; McAlpin et al., 2022). Therefore, we stained the cortical sections (*n* = 6 per group) for the ionized calcium‐binding adapter molecule 1 (Iba‐1) and assessed the morphological characteristics of the microglia. ANOVAs on phenotypic characteristics of microglia indicated a significant effect of treatment on soma size [*F*_(2, 15)_ = 19.98; *p* < 0.0001], number of processes per cell [*F*_(2, 15)_ = 4.49; *p* < 0.05], process length [*F*_(2, 15)_ = 12.60; *p* < 0.001], and number of process branches per cell [*F*_(2, 15)_ = 9.55; *p* < 0.005] (Figure 5a–e). Post hoc comparisons indicated that across all measures, there was no difference between Veh and Dox + ABT. The Dox group exhibited shorter branch length, decreased number of junction, and larger soma size relative to Veh and Dox + ABT groups. While the Dox group exhibited fewer processes relative to Veh, there was no difference between Dox and Dox + ABT. The morphological differences are consistent with previous reports of increased microglial activation due to Dox (McAlpin et al., 2022; Ongnok et al., 2021), which was ameliorated to a certain extent by ABT‐263 treatment.

Vascular dysfunction is associated with Dox treatment, which could lead to BBB disruption (Chunchai et al., 2022). Cortex sections (*n* = 6 per group) were stained for albumin, a peripherally restricted blood protein that enters the brain tissues when there is disruption of the BBB (Figure 5f). There was a significant [*F*_(2, 15)_ = 7.30; *p* < 0.01] difference across groups and post hoc comparisons indicated that the Dox group exhibited an increase in the proportion of cells stained for albumin relative to the other two groups (Figure 5g).

## 3 DISCUSSION

The current study confirmed that Dox impairs episodic spatial and contextual memory in rats as measured on the watermaze and inhibitory avoidance (Ali et al., 2020, 2022; Lal et al., 2023; Liedke et al., 2009). In the current study, we demonstrate that cognitive impairment was linked to peripheral inflammation and oxidative stress, which impaired NMDAR function. In addition, we confirm that many of the Dox‐induced changes are similar to that observed during aging (Bagnall‐Moreau et al., 2019; Gaman et al., 2016; Lopez‐Dominguez et al., 2021; Sun et al., 2022). A novel finding from the current study was that Dox‐induced NMDAR hypofunction through altered redox signaling. A similar redox‐mediated NMDAR hypofunction is observed during aging and linked to cognitive decline (Bodhinathan et al., 2010; Kumar & Foster, 2013; Lee et al., 2014). Finally, most of the Dox‐induced changes were reversed by senolytic treatment with ABT‐263.

There were several similarities in the periphery and the brain, between Dox treatment and aging, which were ameliorated by ABT‐263 treatment. Dox treatment‐induced gene markers of cellular senescence in peripheral tissues (*Cdkn2a*, *Cdkn1a*, *IL‐6*, *Mmp3*, *Tnfsf11*) (Abdelgawad et al., 2023; Lopez‐Dominguez et al., 2021; Sun et al., 2022) and, similar to aging, these markers were reduced by senolytic treatment (Budamagunta et al., 2023). Furthermore, Dox treatment increased systemic cytokines/chemokines, which were reduced by ABT‐263 treatment. Several cytokines/chemokines, which were increased in the current study, have been reported to increase with aging and are associated with impaired cognition of aging (Budamagunta et al., 2023; Scheinert et al., 2015; Serre‐Miranda et al., 2020; Speisman et al., 2013; Yegla & Foster, 2019). However, several differences between aging and Dox treatment are likely important when considering Dox treatment as a model of aging. Cognitive deficits may arise due to a variety of cytokines/chemokines. Due to differences in cell‐specific senescence, the manner in which senescence is induced, and dose relevant Dox‐induced damage, there are likely differences in the level and specificity of plasma cytokines/chemokines associated with aging, inflammatory diseases, and Dox treatment (Coppe et al., 2010). Similarly, the effectiveness of different senolytic interventions will depend on their dose and target cell type. In this regard, most of the Dox reactive cytokine/chemokines were ameliorated by ABT‐263 treatment in the current study.

The release of SASP factors from peripheral tissue likely contributed to observed markers that characterize the brains of aged animals with impaired episodic spatial and contextual memory. These cytokines can disrupt the BBB and induce morphological characteristics of activated microglia. Similar to ABT‐263 treatment of aging animals (Budamagunta et al., 2023), ABT‐263 was effective at preserving the BBB and mitigated morphological changes of microglial soma size, loss of branches, and loss of branch length.

Dox treatment increases plasma cytokines, which enter the brain to drive redox signaling (Aluise et al., 2010b, 2011; Joshi et al., 2010). The current study demonstrates that Dox‐induced redox changes lead to NMDAR hypofunction. A redox‐mediated NMDAR hypofunction is observed early in the course of aging and is associated with impaired spatial episodic memory starting in middle‐age (Kumar & Foster, 2013). Similar to the current study, previous work indicates that memory and NMDAR function can be similarly modulated by treatments that influence systemic inflammation or act on brain redox signaling (Barter et al., 2021; Bean et al., 2015; Budamagunta et al., 2023; Kumar, Rani, et al., 2018; Lee et al., 2014). Thus, the Dox treatment protocol employed in the current study may provide a good model for investigation of the early markers of cognitive aging, including senescent neurophysiology (Foster, 1999, 2019) and associated deficits in episodic memory, which depends on NMDAR function (Foster, 2012).

One previous study employed young mice (3–4 months, 3 mice per group) to examine the effects of Dox on the brain transcriptome (Cavalier et al., 2021). This study found only modest changes in gene expression (~100 total genes) induced by Dox treatment, including decreased expression of genes for nervous system development. We confirmed that Dox treatment decreased expression of genes for nervous system development, including genes linked to neurogenesis (Janelsins et al., 2010; McElroy et al., 2020; Park et al., 2018; Rendeiro et al., 2016; Usmani et al., 2023). Nevertheless, analyses of gene enrichment did not reveal large changes in other biological processes normally associated with aging (Barter et al., 2020; Budamagunta et al., 2023; Ianov, De Both, et al., 2017; Smith et al., 2020; Zeier et al., 2011). Although genes linked to the immune response, oxidative stress, and cellular senescence were significantly upregulated in the Dox group, the number of genes was below the level needed to be considered a significant cluster. The difference in the brain's transcriptional response to Dox treatment, relative to aging, may relate to the transcriptional resilience of young animals in the face of systemic inflammation (Barter et al., 2020) or to the duration of inflammation and age‐related epigenetic changes that contribute to altered gene expression (Barter & Foster, 2018; Ianov, Riva, et al., 2017; Rani et al., 2022; Sinha et al., 2021).

Dox‐induced cognitive impairment is thought to result from peripheral toxic effects and subsequent downstream brain changes (Ren et al., 2019), due in part to the limited ability of Dox to enter the brain (He et al., 2022; Vogler et al., 2011). The results of the current study are consistent with the idea that brain changes were due to peripheral cellular senescence and the release of SASP factors, which enter the brain to alter brain transcription, BBB integrity, microglial activation, synaptic function, and cognition (Budamagunta et al., 2023). Similarly, ABT‐263 exhibits limited brain access (Yamaguchi & Perkins, 2012). Nevertheless, ABT‐263 was able to ameliorate most of the effects of Dox. Thus, similar to aging, ABT‐263 appears to ameliorate brain changes and preserve cognition by reducing peripheral cellular senescence and the release of SASP factors (Budamagunta et al., 2023).

## 4 CONCLUSION

Together these results point to increased peripheral inflammation, associated with senescence of peripheral cells, contributing to brain changes that underlie chemotherapy‐induced cognitive impairment. Thus, it is likely that much of the influence of Dox treatment was mediated by peripheral changes, particularly the increase in systemic cytokines, which then influence synaptic memory mechanisms through oxidative stress.

## 5 EXPERIMENTAL PROCEDURES

### 5.1 Animals

Procedures and experiments pertaining to animals have been reviewed and approved by the Institutional Animal Care and Use Committee (IACUC) of the University of Florida. All the procedures and experiments involving animals were in accordance with the guidelines set forth by the United States Public Health Service Policy on Humane Care and Use of Laboratory Animals. This study utilized male Fischer 344 rats (6 months old), obtained from the breeding colonies of the National Institute on Aging. The animals were maintained in a reverse cycle 12:12 h light/dark schedule and provided with ad libitum access to food and water.

### 5.2 Treatments

Rats were allowed to acclimatize to their new animal facility and the reverse light cycle schedule for at least 10 days before the initiation of any procedure. For this study, rats (*n* = 49; ~6 months old) were divided into three groups of which one received Dox treatment (Dox; *n* = 13), another received a senolytic treatment of ABT‐263 in addition to the Dox treatment (Dox + ABT; *n* = 14). The third group received vehicle treatment (Veh; *n* = 22) and was part of an ongoing study to examine the effects of senolytic treatment on brain aging and cognitive decline (Budamagunta et al., 2023). For the two groups receiving Dox treatment, 2 mg/kg dose of Dox was administered once a week for 4 weeks while the group receiving the senolytic treatment received a 12 mg/kg dose of ABT‐263 for 5 consecutive days every other week (as depicted in Figure 1a). Dox was dissolved in saline and was administered intraperitoneally, while ABT‐263 was dissolved in a vehicle formulation containing 60:30:10 ratio of Phosal 50 PG, PEG400, and ethanol, respectively, and administered by oral gavage (Budamagunta et al., 2023). We monitored body weight over the 4 weeks of treatment for all Dox‐treated animals (Dox = 13, Dox + ABT = 14), as well as for a subset of vehicle‐treated animals (Veh = 14) (Figure 1b).

### 5.3 Behavior testing

#### 5.3.1 Cue discrimination task

All animals (*n* = 22 for Veh, *n* = 13 for Dox, and *n* = 14 for Dox + ABT) were behaviorally characterized. Two weeks after the last dose of Dox and 1 week after the last senolytic treatment, animals were tested on the cue discrimination task, as previously described (Barter et al., 2021; Budamagunta et al., 2023; Foster & Kumar, 2007; Guidi et al., 2014; Kumar & Foster, 2013; Kumar, Rani, et al., 2018). Briefly, a black circular, 1.7 M diameter, water tank within a well‐lit room was surrounded by a black curtain. The temperature of the water was maintained between 27°C and 28°C. An escape platform, roughly 1 cm above the water level, held a white visual cue. Noldus EthoVision software was used to record and process data from the trials. Before the testing, rats were separated into individual cages. After 20 min of acclimatization to the new cages, the animals were habituated to the pool by letting them swim freely for 30 s. Behavioral training consisted of five training blocks of three trials each and the entirety of cue discrimination training was completed in 1 day. The intertrial interval was 20 s and the inter‐block interval was 20 min. At the end of each block, the animal was returned to its cage which was placed under warmed air to prevent hypothermia. The location of the escape platform and the starting location were changed randomly with each trial. Each trial was limited to 60 s and if rats failed to escape, they were gently guided to the platform.

#### 5.3.2 Spatial discrimination task

Three days after the cue discrimination training, animals were trained on the 1‐day spatial version of the water maze to assess their ability to use the distally placed spatial cues to remember and navigate to the location of the submerged platform (Barter et al., 2021; Budamagunta et al., 2023; Foster & Kumar, 2007; Guidi et al., 2014; Kumar & Foster, 2013; Kumar, Rani, et al., 2018). Bright and contrasting objects were placed on all four sides of the pool to act as distally located spatial cues. The escape platform was submerged 1 cm below the water surface and its location was fixed throughout the duration of the spatial discrimination training. The training consisted of five blocks of three trials per block and the start location for each trial was changed randomly for each trial. Rats were given 60 s to find the location of the platform and if they failed to find the platform within the 60 s, they were gently guided to the platform. The intertrial interval was 20 s and the inter‐block interval was 20 min. At the end of each block, the rats were returned to their cage, which was placed under warmed air to prevent hypothermia.

At the end of the fifth block, an acquisition probe trial was performed. The platform was removed from the pool and each rat was started from the quadrant opposite to the goal quadrant, which previously held the platform and was allowed to swim freely for 60 s. After the end of the acquisition trial, the rats were provided a refresher block of three training trials with the platform replaced back in the goal quadrant. The rats were then returned to their home cages and 24 h after the spatial training, the rats were once again tested on a retention probe trial where the platform was removed from the pool and the rat was started from the quadrant opposite the original goal quadrant and allowed to swim for 60 s. To quantitatively assess the performance on the probe trials, discrimination index (DI) scores were calculated using the formula [(time spent in goal quadrant − time spent in opposite quadrant)/(time spent in goal quadrant + time spent in opposite quadrant)].

#### 5.3.3 Inhibitory avoidance

Seven days after the conclusion of the spatial water maze training, an inhibitory avoidance test was conducted to further assess learning and memory retention, based on the protocols established previously (Budamagunta et al., 2023; Foster & Kumar, 2007; Speisman et al., 2013). In short, an inhibitory avoidance apparatus (Coulbourn Instruments, Allentown, PA) comprising of two compartments connected by an automatic door was used for this test. One of the chambers was lit by a light while the other chamber was maintained dark. On the training day, one rat at a time was put into the light chamber and was allowed to acclimatize for 90 s. The connecting door was programed to automatically open at 90 s, allowing the rat to access the dark chamber. The rat was then given 10 min to enter the dark chamber and once all four paws of the rat crossed over to the dark chamber, the automatic door was shut and the rat was given a relatively mild electric shock (0.21 mA) for 3 s. This usually elicits a jumping or rapid movement response, which confirms the rat received an electric shock. Five seconds later, the rat was removed from the chamber and returned to its home cage.

On the testing day (24 h after the training trial), rats were once again placed in the light chamber and allowed to acclimatize for 90 s before the connecting door opened. The rats were then given 10 min to reenter the dark chamber at the end of which the rats were returned to their home cage. Their latency to re‐enter the dark chamber was recorded and was used to assess the spatial context dependent, unconditioned stimulus associated, memory retention of the rats.

#### 5.3.4 Grip strength test

For a subset of animals (*n* = 14 for Veh, *n* = 13 for Dox, and *n* = 14 for Dox + ABT), grip strength was determined as described previously (Carter et al., 2009; Cui et al., 2009; Kumar et al., 2012; Zhou et al., 2011). Briefly, grip strength was assessed using an automated grip strength meter by sensing the peak amount of force an animal applies in grasping the pull bar assembly (Columbus Instruments, Columbus, OH, USA). The rat was handheld by the experimenter using assembly (Columbus Instruments, Columbus, OH, USA). For each measurement, the rat's forelimbs were gently placed on the bar, the animal grabbed the bar (a reflex response in rodents), and was then drawn along a straight line leading away from the sensor. The rat released the pull bar at some point and the maximum force attained was stored on the digital display. The mean force (grams) was calculated over three trials, separated by 2–4 min, and was divided by body weight.

### 5.4 Electrophysiological experiments

Two weeks after the final senolytic treatment, rats were killed, and electrophysiological assays were performed on rat hippocampal sections as described previously (Bodhinathan et al., 2010; Guidi et al., 2015; Kumar, 2015; Kumar et al., 2009, 2019; Kumar & Foster, 2013; Kumar, Rani, et al., 2018). Briefly, rats (Veh = 4, Dox = 5, Dox + ABT = 4) were anesthetized using isoflurane before they were decapitated using a guillotine. The whole brain was then harvested and briefly incubated (~30 s) in a beaker containing prechilled, ice‐cold calcium‐free artificial cerebrospinal fluid (aCSF in mM: NaCl 124, KCl 2, KH_2_PO_4_ 1.25, MgSO_4_ 2, CaCl_2_ 0, NaHCO_3_ 26, and glucose 10). Both hippocampi were then harvested and ~400 μM sections cut parallel to the alvear fibers. These slices were then transferred to the interphase recording chamber where they were incubated (~2 h) in standard aCSF (in mM: NaCl 124, KCl 2, KH_2_PO_4_ 1.25, MgSO_4_ 2, CaCl_2_ 2, NaHCO_3_ 26, and glucose 10), which was continuously oxygenated. The temperature of the aCSF was maintained at a temperature of 30° ± 0.5°C and a pH of 7.4. Glass micropipette electrodes filled with aCSF were used to record excitatory post synaptic field potentials (fEPSPs). The pipette tip was positioned about 1 mm away from the stratum radiatum of CA1 sub region of the hippocampus before evoking 0.033 Hz field potentials through 100 μs pulses of diphasic stimuli. Using a differential AC amplifier and an axoclamp‐2A, the signals from the hippocampal slices were amplified and filtered between 1 Hz and 1 kHz. Input–output curves for total fEPSP were generated by inputting increasing intensities of stimulation.

Following taking input/output curve for total synaptic potentials, the NMDAR‐fEPSPs were isolated as previously described (Guidi et al., 2015; Kumar, 2015; Kumar, Rani, et al., 2018). Briefly, the hippocampal slices were incubated for ≥60 min in aCSF containing 0.5 mM magnesium (Mg2+), 30 μM 6,7‐dinitroquinoxaline‐2,3‐dione (DNQX) and 10 μM picrotoxin (PTX) to isolate NMDAR‐mediated synaptic response. Following isolation of NMDAR EPSP, input–output curve for NMDAR‐EPSP were generated by applying increasing stimulation intensities.

Previous work indicates that a decrease in NMDAR function can result from an increase in oxidative stress associated with inflammation (Kumar et al., 2019; Kumar & Foster, 2013). Therefore, in the present study, we investigated if the reducing agent DTT (0.5 mM) can restore the Dox‐induced decrease in synaptic responses. To examine redox regulation of the NMDAR‐mediated synaptic response, the stimulation intensity was adjusted to elicit a response ~50% of the maximum response, and this baseline, response was recorded 10 min before treatment with DTT for 60 min.

### 5.5 Tissue harvest

Animals were deeply anesthetized with isoflurane and decapitated using a guillotine. Brains were then quickly harvested and rinsed with prechilled saline. Using surgical tools, different parts of the brain were harvested. Placed on a dissection tray on ice, the hippocampus was then carefully dissected into its sub‐compartments namely, CA1, CA3, dentate gyrus (DG), and ventral hippocampus. Once the brain parts were dissected and segregated, the peripheral organs, lung, liver, spleen, and kidney were harvested by making a vertical incision on the ventral surface of the carcass. All the tissues were promptly flash frozen in liquid nitrogen as soon as they were harvested. These tissues were then stored at −80°C until further usage.

### 5.6 Next‐generation RNA sequencing and data analysis

Transcriptional profiles were analyzed in the DG subregion of hippocampus from rats belonging to various groups: young vehicle‐ (Veh; *n* = 10), young Dox‐ (Dox; *n* = 8), young Dox + ABT‐263‐ (Dox + ABT; *n* = 8) treated rats. RNA isolation, library preparation, and transcriptomic sequencing were performed based on the previously published methods (Barter et al., 2019; Ianov et al., 2016; Ianov, De Both, et al., 2017). Briefly, RNA was isolated using RNeasy Lipid Tissue Mini kit (Qiagen, Catalog Number #74804). DNA was eliminated from these samples by using a RNase‐Free DNase Kit (Qiagen, Catalog Number #79254). Using a NanoDrop 2000 spectrophotometer, RNA concentration and purity was measured and RNA integrity number (RIN), from a High Sensitivity RNA ScreenTape in an Agilent 2200 Tapestation system, helped assess the quality and integrity of the isolated RNA. RNA with RIN number greater than 8 was spiked with External RNA Controls Consortium (ERCC) control (Thermo Fisher, Catalog Number #4456740), to assess the quality of the library prepared. Dynabeads mRNA DIRECT Micro Kit (Thermo Fisher; Catalog Number #61021) was used for poly (A) selection of mRNA from the isolated bulk RNA. Using the isolated mRNA, whole transcriptome libraries were prepared with Ion Total RNA‐Seq Kit v2 (Thermo Fisher, Catalog Number #4475936). Ion Xpress barcodes (Thermo Fisher, Catalog Number #4475485) were utilized to enable multiplexed sequencing of multiple libraries. The concentration of the prepared libraries was quantified using Qubit dsDNA High Sensitivity Assay (Thermo Fisher, Catalog Number #32851) and High Sensitivity D1000 ScreenTape in a Tapestation system was used to assess the size distribution of the library. Templates were then prepared on Ion Chef system and sequencing was carried out on an Ion Proton.

For data analysis to obtain the list of differentially expressed genes, Partek Flow server was used. FASTQ files were trimmed and aligned to rat (rn6) genome using STAR. The gene counts were normalized using Median Ratio and any gene with an average number of counts lower than 5 per sample was excluded from the analysis. DESeq2 was utilized to obtain a list of differentially expressed genes. A threshold *p*‐value lower than 0.01 was used as a cutoff to statistically filter genes. Genes that passed this statistical filter were grouped into “upregulated” and “downregulated” genes which were then separately run through NIH Database for Annotation, Visualization, and Integrated Discovery (DAVID) for gene enrichment and functional annotation clustering analysis. This analysis was limited to cellular components, biological process, and molecular function in the “Direct” and “FAT” categories. A Benjamini false discovery rate (FDR) of *p* < 0.05 was used as a threshold to identify significant clusters.

### 5.7 Immunohistochemistry

Cortex tissues (*n* = 6 per group) were fixed in 4% paraformaldehyde for 48 h before being washed with phosphate buffer solution and transferred to 70% ethanol for long‐term storage. These tissues were then embedded in paraffin blocks before making slices of 4 μM thickness. These slices were mounted onto glass slides which were used for immunohistochemistry. Slides were deparaffinized by incubating three times in xylene for 10 min. Deparaffinized sections were then rehydrated by serially incubating the slides in 100%, 95%, 80%, and 60% ethanol for 5 min in each solution. After rinsing with distilled water, the slides were incubated in citrate buffer at 95°C for 45 min. The slides were then rinsed thrice with 1× tris‐buffered saline with 0.1% Tween (TBST) before incubating in 3% hydrogen peroxide for 10 min. The slides were blocked with 10% goat serum for 1 h and then with rabbit anti‐rat albumin/Iba‐1 (diluted 1:250) overnight at 4°C. After being washed three times with 1× TBST for 3 min each, the slides were incubated with goat anti‐rabbit secondary antibody conjugated with HRP for 90 min. The slides were then washed thrice with 1× TBST for 3 min each and were incubated with a solution containing DAB and hydrogen peroxide for 90 s. The slides were then washed with water before incubating with hematoxylin solution for 20 s. The slides were washed again under running water before being dehydrated by serially incubating in 60%, 80%, 95%, and 100% for 2 min each and xylene for 5 min. The slides were then sealed with mounting media and coverslips. Services from the Molecular Pathology Core at the University of Florida were utilized for the timely completion of the immunobiological staining.

The stained sections were blindly scored based on fixed parameters. For albumin staining, 10 random imaged regions per specimen were chosen and the number of albumin positive cells per 50 total cells were counted in each region (*n* = 60 zones/6 specimen). For Iba‐1, multiple parameters such as the size of soma, average length of the longest arm of the processes and the number of processes per cell were measured using ImageJ. Five random imaged regions per specimen were chosen and 5 cells per region were analyzed (*n* = 150 measurements/6 specimen). Measures were averaged for each animal for statistical analyses.

### 5.8 Plasma cytokine analysis

Blood was harvested in ethylenediaminetetraacetic acid tubes while sacrificing the rats (*n* = 6 per group). Plasma was harvested from the blood by centrifuging the tubes at 1600 × *g* for 10 min at room temperature. The plasma was then flash frozen in liquid nitrogen and stored at −80°C for downstream analysis. These plasma aliquots were then subjected to analysis using Rat Cytokine/Chemokine 27‐Plex Discovery Assay by the Eve Technologies Corporation (Calgary, AB, Canada).

### 5.9 Quantitative polymerase chain reaction (qPCR)

RNA was isolated (*n* = 9 per group) from 30 mg of various tissues using RNeasy mini kit (Cat. No. 74106, Qiagen, Hilden, Germany) and was converted into cDNA using a high‐capacity cDNA reverse transcription kit (Cat. No. 4368813, Applied Biosystems, Foster City, CA, USA) following manufacturer's instructions. Gene expression was then quantified using gene specific primers (Table S1) and fast SYBR green master‐mix (Cat. No. 4385617, Applied Biosystems, Foster City, CA, USA) as per the manufacturer's instructions. GAPDH was used to normalize the expression levels across all samples and the gene expression level in untreated tissues was used as baseline to compare the fold change in expression between groups. Fold change in gene expression was determined using ΔΔCT method.

### 5.10 Statistical analysis

Statistical analyses for measures other than next‐generation sequencing (e.g., behavior, electrophysiology, and plasma cytokines) were performed using Statview software (SAS Institute). Parametric variables were presented as mean ± SEM and nonparametric variables were presented with geometric mean. ANOVAs were employed to examine main effects and interactions. Significant differences were localized using Fischer's PLSD post hoc comparisons (*p* < 0.05). One‐tailed one‐group *t*‐tests (*p* < 0.05) were performed to determine if the DI scores were above that expected by chance (i.e., DI score = 0). Kruskal–Wallis test were employed for inhibitory avoidance (*p* < 0.05), with Mann–Whitney *U* tests to localize differences (*p* < 0.05).

## AUTHOR CONTRIBUTIONS

Vivekananda Budamagunta designed and performed experiments, analyzed data, constructed illustrations, and wrote the manuscript; Ashok Kumar designed and performed experiments, analyzed data, constructed illustrations, wrote manuscript; Asha Rani, Sahana Manohar Sindhu, and Yang Yang performed experiments; Daohong Zhou designed experiments and revised the manuscript; Thomas C. Foster designed the experiments, analyzed data, wrote the manuscript, and constructed illustrations.

## CONFLICT OF INTEREST STATEMENT

Daohong Zhou is an inventor of a patent for the discovery of ABT‐263 as a senolytic for the treatment of senescent cell‐associated diseases and a cofounder and a stockholder of Unity Biotechnology that develops senolytic therapy.

## Supporting information


Table S1.

## Data Availability

All data related with these studies are provided as a Supporting Information, and any other details, as needed, will be provided on request. GEO accession number: GSE236404.
