# Supplementary material for: Senolytic treatment alleviates doxorubicin‐induced chemobrain
Source: Aging Cell. 2024 Jan 15;23(2):e14037. doi: 10.1111/acel.14037 (PMC10861213; doi:10.1111/acel.14037)
Supplement: Supplementary file 1 — Table S1. [file ACEL-23-e14037-s001.docx]

**Supplementary Table S1 List of primers used for qPCR**.

| **Gene** | **Forward Primer** | **Reverse Primer** |
| --- | --- | --- |
| GAPDH | TCACCACCATGGAGAAGGC | GCTAAGCAGTTGGTGGTGCA |
| IL6 | TAGTCCTTCCTACCCCAACTTCC | TTGGTCCTTAGCCACTCCTTC |
| TNFSF11 | ATGCAGGAGAATGAAACAAGCCT | CTGTGGCCCCACAATGTGTT |
| MMP3 | TGGGAAGCCAGTGGAAATG | CCATGCAATGGGTAGGATGAG |
| CDKN2A | TGCAGATAGACTAGCCAGGGCA | CTTCCAGCAGTGCCCGCA |
| CDKN1A | AGAGCCACAGGCACCATGTC | ACAGACGACGGCATACTTTGC |
